# Bile Salts Modulate the Mucin-Activated Type VI Secretion System of Pandemic *Vibrio cholerae*

**DOI:** 10.1371/journal.pntd.0004031

**Published:** 2015-08-28

**Authors:** Verena Bachmann, Benjamin Kostiuk, Daniel Unterweger, Laura Diaz-Satizabal, Stephen Ogg, Stefan Pukatzki

**Affiliations:** Department of Medical Microbiology and Immunology, University of Alberta, Edmonton, Alberta, Canada; Institut Pasteur, FRANCE

## Abstract

The causative agent of cholera, *Vibrio cholerae*, regulates its diverse virulence factors to thrive in the human small intestine and environmental reservoirs. Among this pathogen’s arsenal of virulence factors is the tightly regulated type VI secretion system (T6SS). This system acts as an inverted bacteriophage to inject toxins into competing bacteria and eukaryotic phagocytes. *V*. *cholerae* strains responsible for the current 7^th^ pandemic activate their T6SS within the host. We established that T6SS-mediated competition occurs upon T6SS activation in the infant mouse, and that this system is functional under anaerobic conditions. When investigating the intestinal host factors mucins (a glycoprotein component of mucus) and bile for potential regulatory roles in controlling the T6SS, we discovered that once mucins activate the T6SS, bile acids can further modulate T6SS activity. Microbiota modify bile acids to inhibit T6SS-mediated killing of commensal bacteria. This interplay is a novel interaction between commensal bacteria, host factors, and the *V*. *cholerae* T6SS, showing an active host role in infection.

## Author Summary

The type six-secretion system (T6SS) is a molecular syringe that many Gram-negative pathogens use to kill other bacteria, including commensal bacteria of the human gut. We investigated how the environment of the intestine, specifically commensal bacteria, the mucus lining, and bile affect the T6SS of the bacterial pathogen *Vibrio cholerae*. First, we showed that the mucins, a family of proteins ubiquitously found in the intestine, activate the T6SS thereby allowing *V*. *cholerae* to kill other bacteria. Second, we showed that the magnitude of killing is regulated by bile acids. Certain bile acids produced by the host decrease the killing of bacteria by the *V*. *cholerae* T6SS. Last, we demonstrated that prominent members of the host microbiota metabolize these bile acids that enhance bacterial killing by *V*. *cholerae* into bile acids that diminish the bacterial killing effects of the T6SS. Our study suggests that the gut microbiota is an important first line of defense against bacterial pathogens, and that this line of defense may be impaired in individuals in poor health. Promoting a healthy microbial environment in the gut could play a role in counteracting cholera by reducing the ability of *Vibrio cholerae* to compete in the gut.

## Introduction

Upon entering a human host, a bacterial pathogen is vastly outnumbered by both eukaryotic and prokaryotic cells. Pathogens, including the cholera bacterium *Vibrio cholerae*, overcome this numerical disadvantage with strategies to either avoid or fight the host immune system and microbiota[1,2]. The human pathogen *V*. *cholerae* is a marine organism and the causative agent of the diarrheal disease cholera. *V*. *cholerae* participates in both immune evasion and in predatory behavior towards phagocytic immune cells and competitor prokaryotes [2]. After being ingested, *V*. *cholerae* cells traverse the acidic environment of the stomach and penetrate the mucus layer in the small intestine to reach the epithelial lining. There, *V*. *cholerae* cells multiply and secrete cholera toxin, a protein that interferes with adenylate cyclase activity in epithelial cells to cause watery diarrhea in the host and permits dispersal of the multiplying bacteria. During diarrheal purges, high numbers of *V*. *cholerae* cells are carried out of the host and into the environment [3]. Thus, cholera toxin is crucial for the spread of *V*. *cholerae* after it infects a human host.

*V*. *cholerae* strains lacking cholera toxin are still infectious. They colonize the small intestine, elicit immune responses typical of cholera, and cause mild diarrhea [4]. Thus, *V*. *cholerae* uses cholera toxin to generate severe, watery diarrhea for rapid bacterial dissemination, but relies on additional, and for the most part unidentified, virulence mechanisms to establish and maintain infections. We hypothesize that one such virulence mechanism is the tightly controlled type VI secretion system (T6SS) [1,2,5–7]. The T6SS equips *V*. *cholerae* to inject toxic effectors into target cells [1,2,5–7] of both the same and different species [1,8,9]. The T6SS provides *V*. *cholerae* with the means to cope with eukaryotic predators and prokaryotic competitors in the marine environment, and prokaryotic competitors and host macrophages in the human host [7].

The T6SS has structural homology to the contractile cell-puncturing device of T4 bacteriophages. The current model of the T6SS is a nanotube consisting of stacked hexameric rings of the structural Hcp tube protein, with the nanotube being capped by three VgrG effector proteins (one VgrG1, one VgrG2 and one VgrG3) at the tip the puncturing device. VgrG1 and VgrG3 contain C-terminal extensions with enzymatic activities that target prey cells [2,10]. VgrG proteins can carry out additional functions by binding the PAAR-repeat domain of other effector proteins to attach those effectors to the puncturing device [11]. In addition, the adaptor protein Tap-1 delivers T6SS cargo effectors to the VgrG tip[12,13]. The effector-decorated nanotube is believed to dock onto the cytoplasmic side of a baseplate embedded in the *V*. *cholerae* inner membrane [5,11,14–18]. VipA and VipB proteins then form an outer, contractile sheath around the nanotube. Upon contraction of the outer sheath, the Hcp tube, its VgrG tip and attached effectors are ejected from *V*. *cholerae* cells and enter neighboring target cells, thereby delivering the T6SS effectors [6]. Structural proteins, including the inner membrane protein VasK, stabilize the T6SS apparatus [18,19]. Deletion of *vasK* results in an inability to secrete Hcp and T6SS effectors [2].

A wide variety of *V*. *cholerae* T6SS effectors belong to different classes and differ from strain to strain [5,9,16,20]. All strains analyzed to date have three loci where unique effectors can be found. Pandemic *V*. *cholerae* strains all share a set of three cargo effectors, TseL, VasX, and VgrG3 [5,9,16,20]. We refer to the T6SS of pandemic strains as AAA-T6SS, indicating that they all share the same effectors at the same respective loci. AAA-T6SS effectors enzymatically degrade membrane lipids and the peptidoglycan layer of the prey, or insert themselves into the inner membrane to form a pore [10,16,21]. If neighboring *V*. *cholerae* cells engage in T6SS-mediated competition–a phenomenon termed bacterial dueling [22]–both cells can be killed when they use an active T6SS to puncture each other. To prevent the killing of sister cells, immunity proteins are encoded immediately downstream of the T6SS effectors. Immunity proteins are transported into the periplasm where they bind to their cognate T6SS effector delivered by a neighboring cell, thereby abrogating the activity of the effector [10]. Strains in which the genes for these immunity proteins are genetically removed can be used as reporters for T6SS activation, as loss of immunity results in contact-dependent killing under inducing conditions.

Although all *V*. *cholerae* strains sequenced to date carry the genetic information for the T6SS, not all *V*. *cholerae* strains have an active T6SS under laboratory conditions. For example, the O37 serogroup strain V52 (isolated from a cholera patient in the Republic of the Sudan) has an active AAA-T6SS under laboratory conditions. Pandemic *V*. *cholerae* strains, like the O1 serogroup strain C6706 from Peru, do not express T6SS genes under laboratory conditions [23,24]. Zheng et al. [25] recently showed that the T6SS of C6706 is repressed at low cell densities due to the activity of the quorum-sensing regulator LuxO and the transcriptional repressor TsrA (VC0070). At low cell densities, LuxO is phosphorylated and contributes to the generation of small RNAs. These small RNAs specifically bind mRNA transcripts of the large T6SS cluster, thereby inhibiting the translation of important T6SS structural genes and activators [26]. At high cell density, an unphosphorylated LuxO allows activation of the T6SS in pandemic strains when *tsrA* is disrupted. This permits bacterial cells to engage in T6SS-mediated virulence, indicating that high density is critical for expression of the T6SS pandemic *V*. *cholerae* [25]. The ability to genetically activate the T6SS through *luxO* and *tsrA* deletions establishes that pandemic strains have evolved mechanisms to tightly control a fully functional T6SS [25]. The ability to genetically activate the T6SS through *luxO* and *tsrA* deletions establishes that pandemic strains have evolved mechanisms to tightly control a fully functional T6SS [25].

We currently do not know when and where pandemic *V*. *cholerae* uses the T6SS during its life cycle. Experimental evidence suggests that pandemic *V*. *cholerae* strains de-repress T6SS gene expression during infection of the small intestine of infant mice and of humans [27–29]. More recently, *V*. *cholerae* was shown to engage in T6SS-mediated killing in infant rabbits [27–29]. The biological significance of *in-vivo* T6SS de-repression, however, remains unknown. T6SS de-repression has also been shown in *V*. *cholerae* cells attached to chitin, which *V*. *cholerae* utilizes during colonization of copepods [30]. This activation appears to be coupled with the ability of *V*. *cholerae* to become naturally competent, by allowing *V*. *cholerae* to kill a prey cell and then take up its DNA[31].

During infection of the human host, *V*. *cholerae* is penetrates the mucus layer of the small intestine to make intimate contact with the epithelium and elicit cholera toxin-induced diarrhea [32]. The intestinal epithelium is protected by a thick layer of mucus that consists of a mix of highly glycosylated mucin proteins [33]. Mucins act as a natural barrier against pathogenic and commensal bacteria, with commensals only colonizing the luminal side of the mucus layer [34,35]. Unlike commensal bacteria, many pathogens have evolved strategies to overcome the antimicrobial molecules within the mucin barrier as well as to transverse the mucin barrier [36]. *V*. *cholerae* expresses the mucin-binding factor GbpA to successfully colonize the murine small intestine [37,38] and secretes mucinases HapA and TagA via the type II secretion system [39] to penetrate the mucus layers [40,41].

In addition to mucins, *V*. *cholerae* is exposed to another prominent host factor, bile. Primary bile acids, including cholic acid and its conjugates glycolic acid and taurocholic acid, are synthesized in the liver and released into the small intestine upon hormonal stimulation. Dehydroxylation of these primary bile acids by commensal bacteria in the gastrointestinal tract generates secondary bile acids; these include deoxycholic acid, glycodeoxycholic acid and taurodeoxycholic acid. Yet other commensal bacteria have the ability to deconjugate bile acids, removing the glycine or taurine and returning the conjugated compound to either cholic acid or deoxycholic acid. While some bile acids are excreted with feces, most undergo enterohepatic circulation; that is, they are absorbed from the terminal ileum (the most distal portion of the small intestine adjacent to the large intestine), transported to the liver for conjugation to either glycine or taurine, and hydroxylated to regenerate primary bile acids. The cycle starts over when primary bile acids are returned to the intestine [42].

Actions of both the host and commensal bacteria contribute to the multifaceted composition of bile in the gastrointestinal tract. Experimental evidence is growing that dehydroxylation of primary bile acids can drastically alter virulence factors produced by bacterial pathogens. For example, deoxycholic acid (but not cholic acid) induces virulence gene expression in *Campylobacter jejuni* necessary for its invasion of macrophages [43]. In contrast, taurocholic acid (a hydroxylated bile acid) has been implicated in biofilm dispersal and motility of *V*. *cholerae* as well as in increasing *tcpA* expression [44]. Virulence factors thus can be regulated differently throughout the intestine depending on the bile acids present and, by extension, the presence of different commensal bacteria.

In this study, we investigated the molecular basis for host control of the *V*. *cholerae* T6SS. We discovered that mucins relieve repression of the T6SS in pandemic strains. The T6SS, now de-repressed, can be then down-regulated by deoxycholic acid, a metabolite of cholic acid created by commensal bacteria. Our findings suggest that mucins, prevalent throughout the gut, activate the T6SS whereas bile acids fine-tune T6SS activity.

## Materials and Methods

### Ethics statement

Animal studies used in this study (AUP0000320) were reviewed and approved by the Animal Care and Use Committee–Health Sciences at the University of Alberta. This committee adheres to the policies and standards of the Canadian Council on Animal Care. The University of Alberta’s Animal Welfare Assurance Number is #A5070-01.

### Bacterial strains and culture conditions

Bacterial strains were grown overnight on LB (Luria Bertani) agar plates supplemented with 100 μg/mL streptomycin (Sm) or 100 μg/mL rifampicin (Rif). A derivative of *V*. *cholerae* strain V52 (O37 serogroup; Sm^R^) lacking *hlyA*, *rtxA*, and *hapA* was used as the wild-type strain in all experiments. The *E*. *coli* K-12 strain MG1655 (genotype F^**−**^, **λ** ^**−**^, rph^-1^, Rif^R^) was provided by Tracy Raivio (University of Alberta). *V*. *cholerae* C6706 (O1 serogroup; Sm^R^) and N16961 (O1 serogroup; Sm^R^) were provided by John Mekalanos (Harvard Medical School, Boston MA). Anaerobic bacteria *Bifidobacterium bifidum* (ATCC 15696) and *Bifidobacterium subtile* (ATCC 27683) were obtained from Cedarlane Laboratories, Burlington ON. *Bifidobacterium adolescentis* (ATCC 15703), and *Bacteroides thetaiotaomicron* (ATCC 29148) were gifts from Alain Stintzi (University of Ottawa, Ottawa ON). Anaerobic bacteria were grown on brain heart infusion (BHI) plates or in BHI medium under anaerobic conditions in plastic or metal jars with AnaeroGel sachets (Oxoid; Hampshire, England).

### In-frame deletions and cloning

In-frame deletions of *tsrA* and *luxO* were performed as described in Metcalf et al. [45]. Briefly, a *tsrA* knockout construct was created using the following primers:

A 5´-GGATCCGATTTGCGCTGCTGGTAGGG-3´

B 5´-TCGATTAGCGTTTTTGTAAGGTGGTTAGAGACATGGTG-3´

C 5´-CACCATGTCTCTAACCACCTTACAAAAACGCTAATCGA-3´

D 5´-GGATCCCTGCAAGCTCGCTGTCCC-3´

PCR products resulting from primer combinations A/B and C/D were stitched together by overlapping PCR. The resulting knockout construct was digested with *Bam*HI and cloned into the suicide plasmid pWM91. The *luxO* knockout construct was generously provided by David Raskin (Marian University, Indianapolis IN). C6706Δ*luxO* was used as the parental strain from which *tsrA* was deleted to create the C6706Δ*luxO*Δ*tsrA* double mutant.

### *In vivo* and *in vitro* assays

*In-vivo* competition assays were performed as described previously [46]. Pregnant CD1 mice were purchased from Charles River Laboratories. Animals were treated in accordance with protocols approved by the University of Alberta Animal Care and Use Committee. Briefly, indicated bacterial predator and prey strains were grown on selective LB agar plates overnight. 1 × 10^9^ bacteria were resuspended in a 2.5% sodium bicarbonate buffer, mixed at a 1:1 ratio, and fed via oral gavage into 3-day-old CD1 infant mice. After 16 hours, the pups were sacrificed and their small intestines homogenized in 1 mL phosphate-buffered saline. In parallel, this experiment was performed *in vitro* by adding 50 μL of *V*. *cholerae* mixture (in 2.5% sodium bicarbonate buffer) to 5 mL LB. Cultures tubes were rolled overnight at 37°C. Serial dilutions of the *in-vitro* and *in-vivo* samples, and the bacterial inoculum, were plated on LB agar plates supplemented with Sm and 5-bromo-4-chloro-3-indolyl-β-D-galactopyranoside (X-gal) to count predator and prey strains. The competitive index was calculated by taking the input ratio (mutant/wild-type) and dividing it by the output ratio (mutant/wild-type).

### Killing assays

Killing assays were performed as described previously [1]. Briefly, indicated bacterial strains were grown overnight on LB agar plates with appropriate antibiotics. Prey and predator were mixed at a 10:1 or 1:1 ratios with titers normalized by OD_600_ readings. Mixtures were spotted on LB agar plates with indicated supplements and incubated for 4 h at 37°C. Bacteria were harvested and serial dilutions of rifampicin-resistant prey or streptomycin-resistant predator were selectively grown on plates overnight. Sodium deoxycholate, sodium cholate, taurodeoxycholate, glycodeoxycholate, taurocholate, glycocholate, and taurine were obtained from Sigma (St. Louis MO). Glycine was obtained from Thermo Fisher Scientific (Waltham MA). Difco™ Bile Salts No.3 was obtained from BD (Mississauga ON). For assays of anaerobic bacteria, indicated bacteria were spotted on LB agar plates supplemented with 1.2 mM bile acids and incubated for 2 days under anaerobic conditions. Bacteria were scraped from the plates and plates were treated for 15 min with chloroform and incubated for an additional 15 min at 37°C [47]. This procedure ensured that all anaerobic bacteria were dead, and only their metabolites remained. Killing assays were performed on these plates.

### Immunoblots

Overnight cultures of indicated bacterial strains were diluted in LB broth supplemented as indicated and grown to mid-logarithmic phase (OD_600_ ~ 0.6). Samples were subjected to SDS-PAGE and analyzed by western blotting using a mouse monoclonal antibody against DnaK (Stressgen Bioreagents, Victoria BC), anti-FLAG M2 (Sigma, St. Louis MO), or a rabbit polyclonal antibody against Hcp [7]. For detection, secondary antibodies goat anti-rabbit-horseradish peroxidase and goat anti-mouse-horseradish peroxidase were used (Santa Cruz Biotechnology, Santa Cruz CA).

### Mucin column assays

Columns contained 500 μl of 3% (wt/vol) bovine submaxillary mucins (Sigma, St. Louis MO) or 3% (wt/vol) of Difco gelatin (BD, Mississauga ON) in Krebs-Ringer Tris buffer [48]. Columns were allowed to settle for 1 h at room temperature. For viability tests, columns were prepared in 1.5 ml reaction tubes. Approximately 1 × 10^8^ mid-logarithmic phase bacteria (20 μL) were loaded on top of each column and incubated for 1 h or 2 h at 37°C. One hour after adding supplementing bile acids/amino acids, colony-forming units (CFUs) were determined by plating serial dilutions on LB agar plates with appropriate antibiotics. For killing tests, columns were prepared in 1.5 ml reaction tubes. Indicated predator and prey strains were loaded on top of each column at a 10:1 ratio and incubated for 2 h at 37°C. CFUs/ml were determined by plating serial dilutions on LB agar plates with appropriate antibiotics.

### RNA isolation, cDNA synthesis, and qPCR

Total RNA was extracted using the TRizol reagent (Invitrogen) according to the manufacturer's instructions. RNA concentrations were determined using a NanoDrop spectrophotometer (Thermo Scientific). 1μg of RNA from each sample was treated with DNase I (Invitrogen), and transcribed into cDNA using the SuperScript III Reverse Transcriptase (Invitrogen). Quantitative real-time PCR (qPCR) was performed with SensiFAST SYBR No-ROX Kit (FroggaBio), using the CFX96 Real-Time System (Biorad). Thermocycling parameters were as follows: 95°C for 2 min, followed by 40 cycles of 95°C for 15 s and 60°C for 1 min, followed by a melting curve. Primers against the different genes of interest were designed using the PrimerQuest software from Integrated DNA Technologies (IDT). Primers were tested for performance in qPCR with a cDNA concentration gradient, and those with slopes between −3.3 and −3.7, efficiency of ∼1.0, and *R* ^2^ of ∼1.0 were used in the qPCR studies (primer sequences used in the study are summarized in Table 1). The expression levels of the different targets in relation to the endogenous 16S rRNA gene control was determined by the 2^−ΔΔCT^ method using the CFX Manager Software (Biorad). The relative quantification (RQ) values of all samples were normalized against the expression of the 16S control for each target.

**Table 1:** Oligonucleotide sequences for qPCR.

Target	Direction	Sequence
16S rRNA gene	F	GTG TAG CGG TGA AAT GCG TAG AG
	R	GCG TGG ACT ACC AGG GTA TCT AAT
*hcp*	F	TGT GAA ATG CCA CAC TGC CAA GAC
	R	GCG TTA ACG TGG TCC CAA GTG ATT
*tseL*	F	GTT AGA GCT AGA GTT TCG GAG TG
	R	GTG GTT TGC GTG TAT GTG TTA G
*vasX*	F	GAG TCA GAA ACT GGG TGG ATT AG
	R	GTG CGA CCT TAT AGC GGA TAT T
*vgrG-3*	F	CTC GTG GTA CAA GCC AAT CA
	R	AGT GAT GTG AGC GGG AAT AAG
*vasH*	F	TAT CTG CCA CAC AGC TCA ATC
	R	CAA GGT GAT CGG ATA CTG GAA TAG

F, forward; R, reverse

### Thin-layer chromatography

Thin-layer chromatography (TLC) was performed as described [49]. Briefly, overnight cultures of *B*. *subtile*, *B*. *bifidum*, *B*. *adolescentis*, or *Bacteroides thetaiotaomicron* were harvested by centrifugation and resuspended in Ringer’s solution. Reaction mixtures were prepared by dissolving indicated concentrations of bile acids in BHI medium. Bacterial suspensions were added in a 1:1 ratio to the reaction mixture; control solutions were incubated without bacterial suspension. After anaerobic incubation overnight at 37°C, samples centrifuged and the supernatant was filtered, lyophilized and residues were redissolved in 1 mL methanol. After centrifugation, 3 μl of supernatant was spotted onto a TLC sheet (Polygram™ SilG precoated with 0.25 mm silica gel; Machery-Nagel, Germany) (stationary phase) and dipped into the mobile phase. The mobile phase consisted of isoamyl acetate, propionic acid, n-propanol, and water (40:30:20:10; Fisher Scientific, Ottawa ON). After the run was completed, TLC sheets were dried for 3 min at 110°C, and bile acids were located by spraying sheets with 10% phosphomolybdic acid in ethanol (Fisher Scientific, Ottawa ON). R_f_-values were determined by dividing the distance traveled by the bile acid by the distance traveled by the solvent front.

### Growth curves

Overnight cultures of *V*. *cholerae* were diluted 1:100 in plain LB broth or LB supplemented with various concentrations of deoxycholic acid, glycine or taurine. OD600 readings were recorded every hour. The resulting ODs were plotted versus time, and the linear portion of the graph was used to calculate a slope as described in Provenzano *et al*. (2000) [50]. This slope was then compared to the bacteria’s slope when grown in plain LB to determine its relative growth rate.

## Results

### T6SS-mediated killing induced *in vivo*

Once inside the host, *V*. *cholerae* has to overcome the host defense of commensal gut bacteria that secrete bacteriocins and compete for nutrients and attachment sites. We do not yet understand how *V*. *cholerae* outcompetes the commensal host flora despite *V*. *cholerae*’s numerical inferiority upon arrival in the gut. We hypothesized that the pandemic O1 strain C6706 establishes an infection by relieving repression of its T6SS to engage in T6SS-mediated competition with the commensal microbiota. To extend recent findings that *V*. *cholerae* turns on T6SS genes and engages in T6SS-mediated dueling in the infant rabbit [27,29], we employed the more accessible infant mouse model in this study. The established mouse model for cholera was previously used to study regulation and anti-eukaryotic activity of the *V*. *cholerae* T6SS [46].

**Fig 1:**
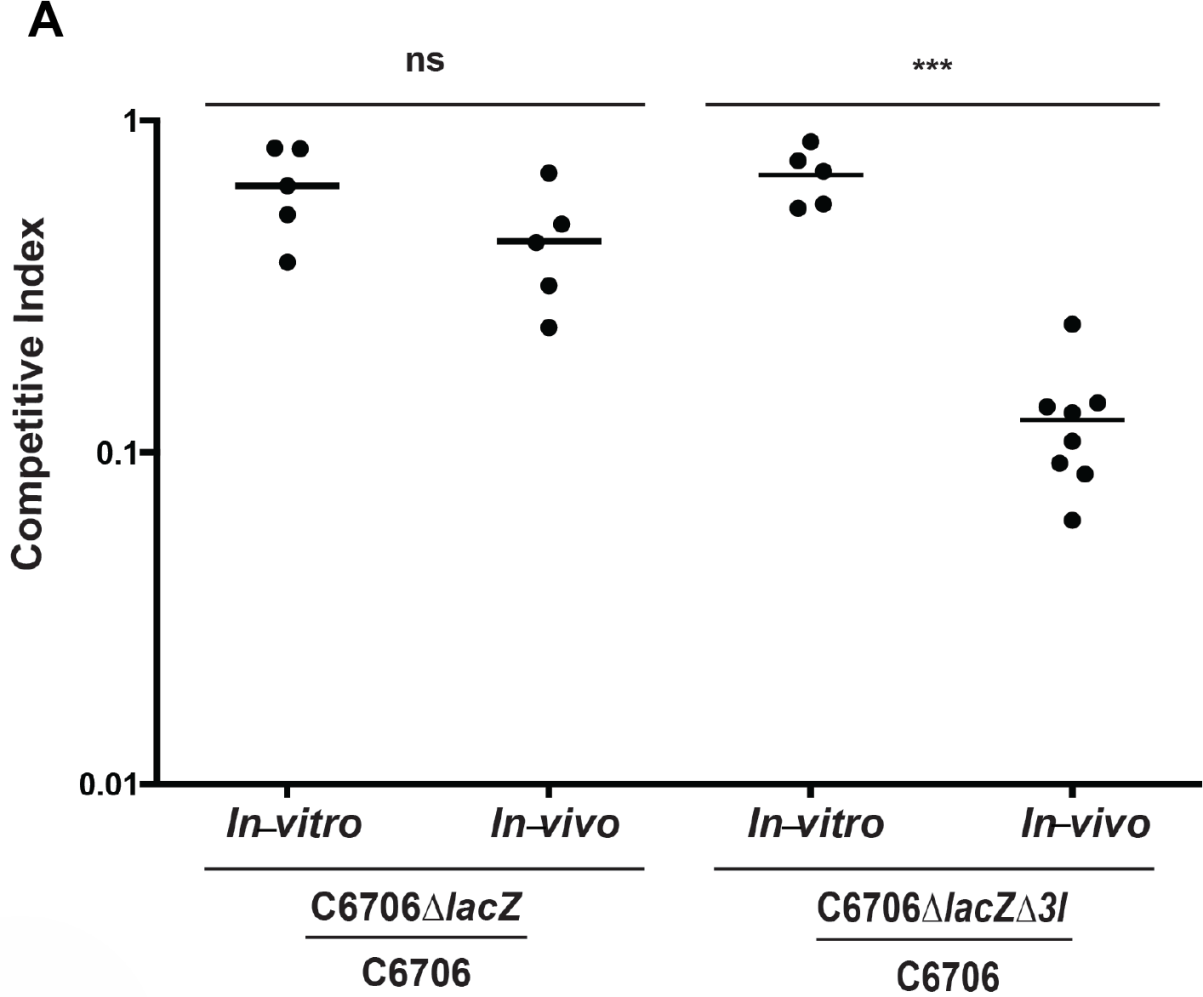
The T6SS of *V*. *cholerae* is functional *in vivo*. The T6SS of *V*. *cholerae* O1 strain C6706 is active in the infant mouse model of infection. *V*. *cholerae* C6706, C6706Δ*lacZ*, and C6706*ΔlacZΔtsiV1-3* were mixed in pairwise combinations and administered to the infant mouse as mixtures via oral gavage. As an *in-vitro* control, 2 mL LB were inoculated with the same bacterial mixtures and grown overnight at 37°C. After a 16-h incubation, the mice were sacrificed and their small intestines were harvested and plated on X-gal plates to count surviving bacteria. The competitive index of the two competing strains is shown on the *y-*axis. Horizontal bars represent the arithmetic mean of one experiment performed with a minimum of 5 mice in each group. Stars indicate statistical significance (unpaired two-tailed Student’s *t*-test), with *** *p* < 0.0005, ns = not significant.

We co-infected infant mice with wild-type C6706 and C6706Δ*tsiV1-3*, which carries in-frame deletions of three T6SS immunity genes. The immunity proteins bind to their cognate effectors. When the immunity proteins are genetically removed, the bacteria are unable to defend themselves from the killing effectors from a T6SS-positive sister cell. T6SS-mediated killing of C6706Δ*tsiV1-3* was therefore used as a read-out for T6SS activity. The immunity proteins of C6706 bind the effectors TseL, VasX and VgrG3. The relative viability of wild-type C6706 remained the same in both *in vivo* and *in vitro* assays; in contrast, we observed a significant drop in surviving *V*. *cholerae* C6706Δ*tsiV1-3 in vivo* compared to *in vitro* (Fig 1). To determine if the reduced colonization *in vivo* of *V*. *cholerae* C6706Δ*tsiV1-3* was due to a T6SS-independent colonization defect associated with the removal of three immunity genes, we performed an additional co-infection experiment with C6706Δ*vasK* and C6706Δ*vasK*Δ*tsiV1-3*. In this scenario, neither strain encodes a functional T6SS. Co-infection with these two strains resulted in no difference in total bacterial numbers (S1A Fig) and no difference in survival for either strain between the *in vivo* and *in vitro* experiments (S1B Fig). This indicates that reduced colonization by C6706Δ*vasK*Δ*tsiV1-3* in the presence of C6706 *in vivo* as shown in Fig 1 is not due to a T6SS-independent colonization defect, but rather a consequence of succumbing to T6SS-mediated toxicity. These results confirm *in-vivo* T6SS activity in the infant mouse.

The observation that *V*. *cholerae* uses its T6SS in infant mouse and rabbit models prompted us to identify host cues responsible for T6SS activation. One feature of the small intestine is its low level of oxygen that fluctuates between anaerobic and microaerobic conditions [51]. To determine if *V*. *cholerae’s* T6SS is activated under anaerobic conditions, we performed a killing assay in which we mixed *V*. *cholerae* C6706 with *E*. *coli* prey under aerobic and anaerobic conditions. Anaerobic conditions did not affect *E*. *coli* viability, as the *E*. *coli* grew equally well under anaerobic and aerobic conditions in the presence of V52Δ*vasK* (a V52 mutant in which the T6SS is disabled by deletion of the T6SS gene *vasK*) (S1C Fig). When *E*. *coli* was mixed with C6706, no T6SS killing occurred, suggesting that C6706 maintains a repressed T6SS under anaerobic condition. To determine if an anaerobic environment permits T6SS-mediated virulence per se, we repeated the assay with V52 as the predator, because this strain employs a constitutively active T6SS. We observed that T6SS-mediated killing still occurs in the absence of oxygen, as shown in S1C Fig. This suggests that pandemic *V*. *cholerae* senses signals other than the absence of oxygen to activate the T6SS.

### Mucins relieve repression of a functional T6SS in pandemic *V*. *cholerae* strains

**Fig 2:**
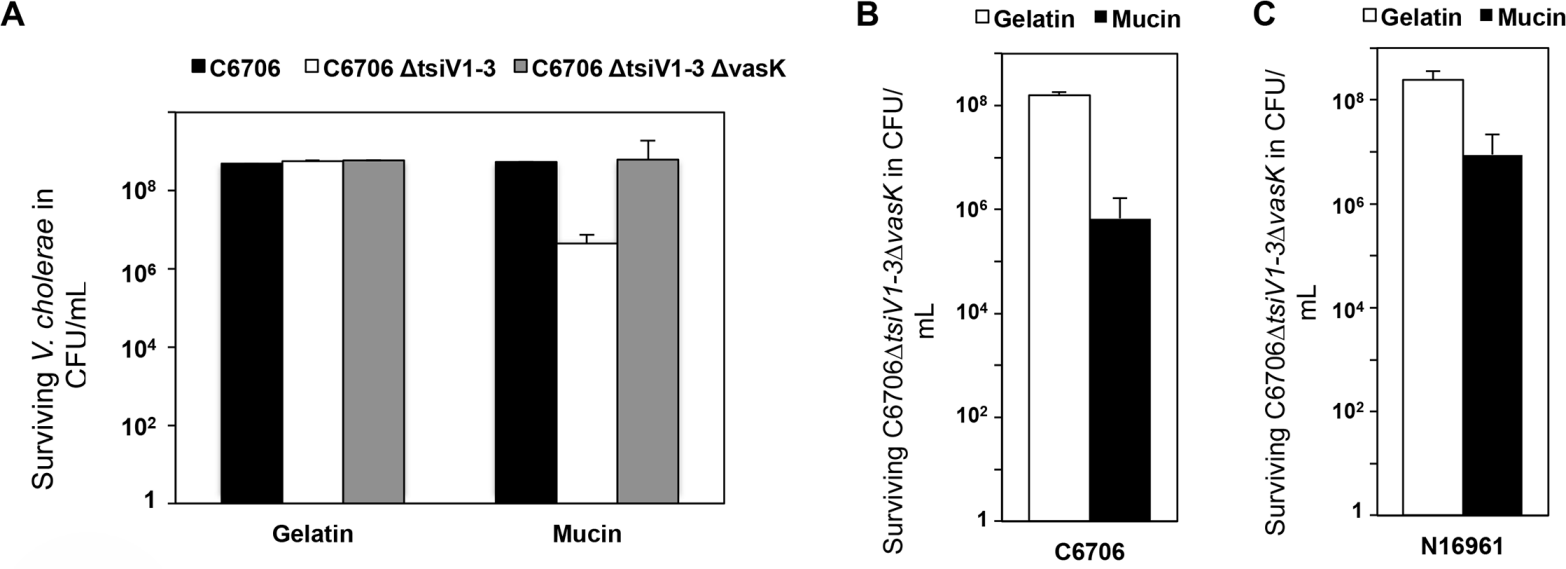
*V*. *cholerae* O1 strains have an activated T6SS in the presence of mucins. **(A)** Survival assay of *V*. *cholerae* C6706 mutants on mucins. 1 × 10^8^ *V*. *cholerae* C6706, C6706Δ*tsiV1–3* (lacking all three immunity genes), or C6706Δ*tsiV1–3*Δ*vasK* (lacking all three immunity genes and the T6SS gene *vasK*) were loaded separately on columns containing 3% mucins or 3% gelatin. After incubation for 2 h at 37°C, eluents were collected, and serial dilutions were plated on selective LB agar plates. Surviving numbers of *V*. *cholerae* bacteria were plotted as CFUs. Bars represent mean values ± SD of two independent experiments done in duplicate. **(B)** Intraspecies killing by *V*. *cholerae* C6706 on mucin columns. Killing assays were performed by mixing predator C6706 and prey C6706Δ*tsiV1–3*Δ*vasK* at a ratio of 10:1 and loading them together on gelatin or mucin columns. After incubation for 2 h at 37°C, cells were collected and serial dilutions were plated on LB agar plates. Bars show mean values ± SD of two independent experiments done in duplicate. **(C)** Intraspecies killing by *V*. *cholerae* N16961 on mucin columns. Killing assays were performed by mixing predator N16961 and prey C6706Δ*tsiV1–3*Δ*vasK* at a ratio of 10:1 and loading them together on either gelatin or mucin columns. After incubation for 2 h at 37°C, eluents were collected and serial dilutions were plated on LB agar plates. Bars show mean values ± SD of two independent experiments done in duplicate.

Although the T6SS of the *V*. *cholerae* C6706 strain is inactive under laboratory conditions, RNASeq data suggest that T6SS genes are expressed *in vivo* [27,52]. The T6SS has been demonstrated to be functional and to mediate interbacterial interactions in the infant rabbit [29] and mouse (Fig 1). However, we do not yet know how the repression of the T6SS is relieved in the host. The mucus layer is the first site of contact for *V*. *cholerae* in the small intestine, therefore we hypothesized that mucins (the main protein components of the mucus layer) relieve the repression of the *V*. *cholerae* T6SS. Interaction with mucins increases *V*. *cholerae* motility and successful colonization of the murine small intestine [37,53]. To determine if mucins influence the *V*. *cholerae* T6SS, we exposed the pandemic O1 strain C6706 or an isogenic mutant lacking three immunity genes (C6706Δ*tsiV1-3*) to a 3% mixture of mucins or gelatin (which possesses the same viscosity as mucins) as a negative control (Fig 2A). In *V*. *cholerae* strains such as C6706, N16961, and V52, the immunity proteins TsiV1, TsiV2, and TsiV3 confer resistance to the T6SS toxins TseL, VasX, and VgrG3, respectively [5,16,20]. We compared numbers of bacteria recovered from either mucin or gelatin columns loaded with one of these three strains. Viable C6706 bacteria were recovered in equal numbers from gelatin or mucin columns, in contrast to the recovery of ~ 1 log fewer viable C6706Δ*tsiV1-3* bacteria from mucin columns than from gelatin columns (Fig 2A). With all three immunity genes missing, *V*. *cholerae* cells are unable to defend themselves against T6SS-mediated killing by siblings [54]. Thus, the failure of C6706Δ*tsiV1-3* to survive in the presence of mucins suggested that mucins relieve the T6SS repression, resulting in a functional T6SS in pandemic strains. We confirmed that death of C6706Δ*tsiV1-3* in the presence of mucins is due to sibling-mediated killing and not suicide due to the absence of immunity proteins by genetically disabling its T6SS. An immunity mutant with a disabled T6SS, C6706Δ*tsiV1-3*Δ*vasK*, survived in mucins (Fig 2A) unless challenged with parental C6706 (Fig 2B), confirming that the viability defect is T6SS-dependent. Furthermore, there is no growth defect of any of these strains as demonstrated by growth curves of C6706 in LB (S2 Fig). These experiments show that mucins are sufficient to coordinate assembly of a functional T6SS, and confirm that immunity genes are required for protection against sibling-mediated killing.

To determine if this mucin-dependent phenomenon is a general characteristic of 7th pandemic strains, we tested the effect of mucins on an additional El Tor strain, N16961, that was independently isolated and represses the AAA-T6SS under laboratory conditions [1]. Similarly to C6706, N16961 killed C6706Δ*tsiV1-3*Δ*vasK* only in the presence of mucins (Fig 2C). Although C6706 and N16961 differ in their degree of T6SS killing activity, this experiment shows that mucins relieve the repression of the T6SS in these two 7^th^ pandemic O1 strains that utilize the same set of T6SS effectors [9].

### Bile acids influence the T6SS function of pandemic *V*. *cholerae* in the presence of mucins

**Fig 3:**
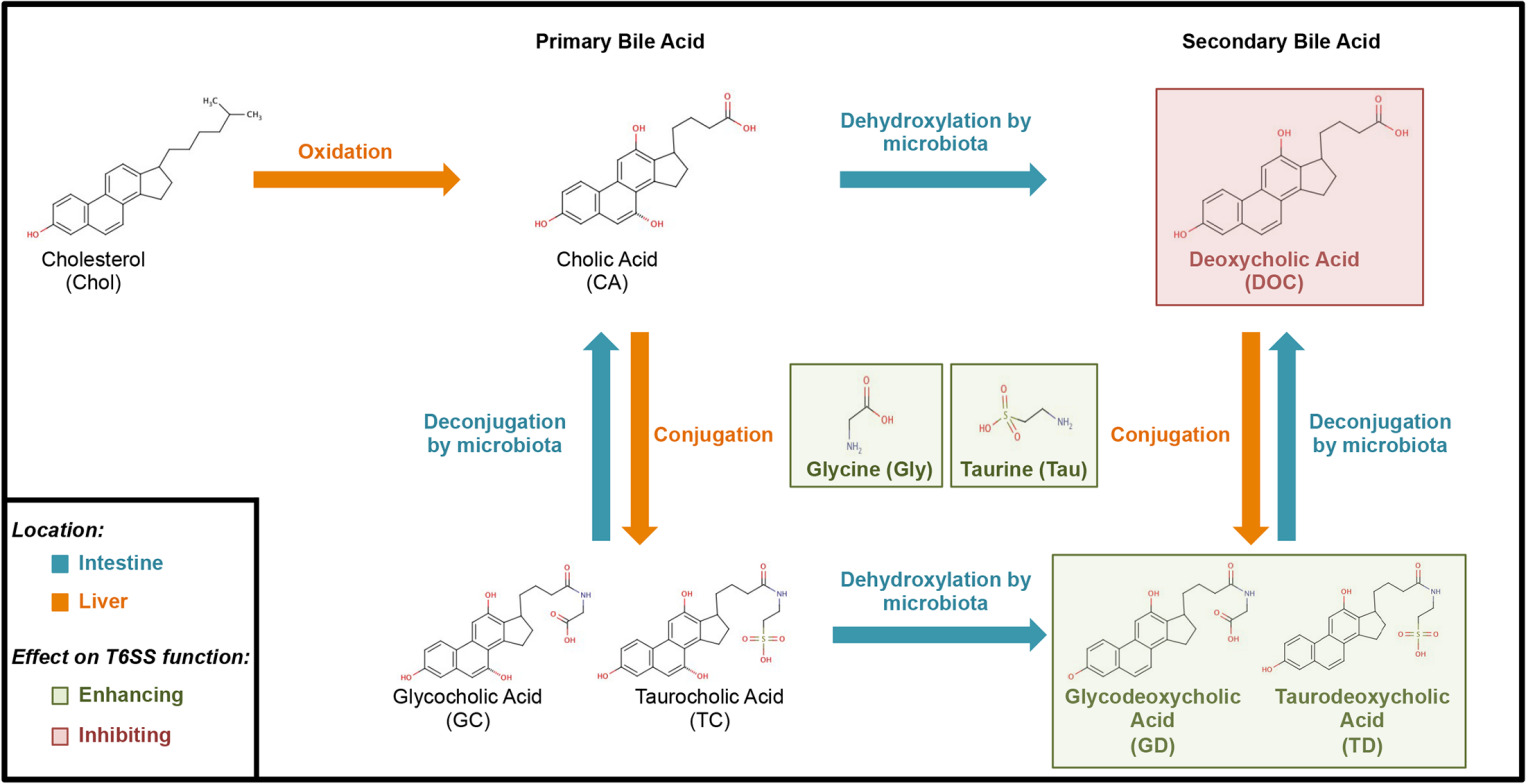
Bile acid metabolism. Flow diagram showing the metabolism and fate of bile acids. In the liver (orange arrows), cholesterol is enzymatically converted to primary bile acids such as cholic acid. After conjugation of taurine or glycine to cholic acid by hepatocytes, the resulting glycocholic acid or taurocholic acid are stored in the gall bladder until released into the small intestine in response to food ingestion. In the small intestine (blue arrows), several commensals deconjugate and/or dehydroxylate bile acids, producing unconjugated deoxycholic acid and cholic acid. Alternatively, glycholic acid or taurocholic acid can be dehydroxylated to the conjugated secondary bile acids glycodeoxycholic acid or taurodeoxycholic acid. In a process called enterohepatic circulation, unconjugated bile acids are excreted with the feces or reabsorbed from the ileum back into the liver. Depending on the types of microbiota and nutrition ingested, bile acid composition may vary throughout the intestines. Therefore, the inhibitory effect identified in this study (red) of deoxycholic acid and the enhancing effect (green) of glycodeoxycholic acid or taurodeoxycholic acid on the T6SS of *V*. *cholerae* may vary depending on the microbiota present.

Bile is abundantly present in the gastrointestinal tract and consists of water, a mixture of different primary and secondary bile acids, fats, inorganic salts, pigments, and immunoglobulins [55]. In response to bile, *V*. *cholerae* regulates the expression of the principle virulence factors cholera toxin and toxin-coregulated pilus [56–58]. We investigated whether the bile metabolism pathway (Fig 3) generates bile acids that modulate T6SS activity. Recent work by the Mekalanos group showed that with the LuxO/TsrA repressing circuit disabled, pandemic *V*. *cholerae* employs a hyperactive T6SS and causes severe inflammation in the small intestine of experimental animals [25]. We hypothesized that T6SS gene expression might be transient and tightly controlled by host factors in addition to mucins, restricting the activation of the *V*. *cholerae* T6SS to specific times and/or locations during infection of the small intestine.

**Fig 4:**
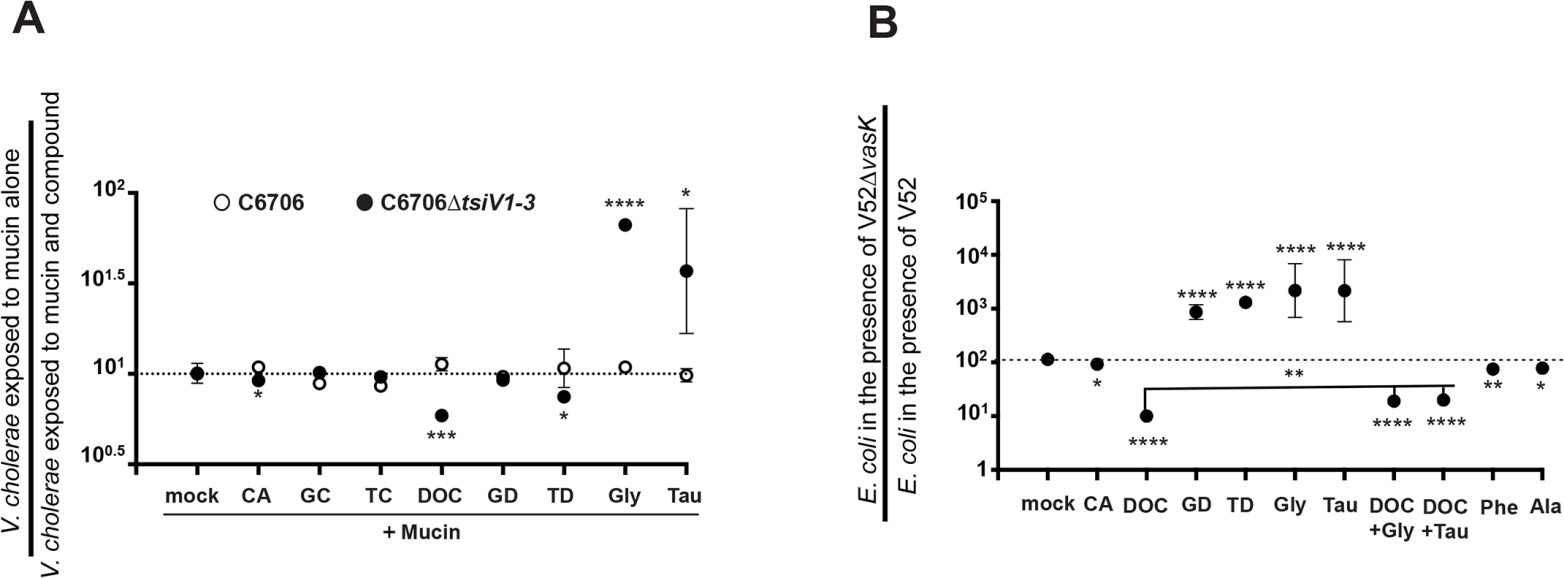
Deoxycholate diminishes T6SS function. **(A)** Bile acids affect a mucin-activated T6SS of *V*. *cholerae* C6706. 1 × 10^8^ *V*. *cholerae* C6706 or C6706Δ*tsiV1–3* were loaded separately on columns containing 3% mucins. After incubation for 1 h at 37°C, either cholic acid (CA), glycocholic acid (GC), taurocholic acid (TC), deoxycholic acid (DOC), taurodeoxycholic acid (TD), glycodeoxycholic acid (GD), glycine (Gly), or taurine (Tau) were added to the mucin columns at a final concentration of 1.2 mM each. After incubation for 2 h at 37°C, cells were collected, serial dilutions were plated on LB agar plates, and surviving numbers of C6706 or C6706Δ*tsiV1–3* were plotted. Bars show mean values ± SD of two independent experiments done in duplicate. **(B)** Individual bile acids affect the T6SS of *V*. *cholerae* V52. Predator *V*. *cholerae* V52 or V52Δ*vasK* were mixed at a 10:1 ratio with prey *E*. *coli* MG1655 and spotted on LB agar plates supplemented with 1.2 mM cholic acid (CA), glycocholic acid (GC), taurocholic acid (TC), deoxycholic acid (DOC), glycodeoxycholic acid (GD), taurodeoxycholic acid (TD), glycine (Gly), or taurine (Tau) for 4 h at 37°C. CFUs were counted after incubation of serial dilutions of eluent on LB agar plates overnight. The killing index was calculated by the ratio of surviving prey in the presence of V52 versus attenuated V52. The graph gives mean values ± SD of two experiments done in duplicate. A Student’s *t*-test was performed for significance, with *p < 0.05, ** *p* < 0.005, *** *p* < 0.0005, **** *p* < 0.0001.

As bile acids diffuse through the mucus layer during fat absorption [59], *V*. *cholerae* is likely to be exposed to mucins and bile in the same locale and at the same time. Thus, we tested whether bile acids can modulate the mucin-activated T6SS of *V*. *cholerae* O1 serogroup strain C6706. As shown in Fig 4A, wild-type C6706 and C6706Δ*tsiV1–3* (as an indicator strain for T6SS activity) were grown either in the presence of gelatin, mucins, or mucins supplemented with cholic-, glycocholic-, taurocholic-, deoxycholic-, glycodeoxycholic-, or taurodeoxycholic acids, or glycine or taurine (see Fig 3 for metabolic context of each bile salt). The only compounds that enhanced mucin-induced T6SS killing of C6706Δ*tsiV1-3* kin cells were glycine and taurine (Fig 4A). Deoxycholic acid, but not its precursor cholic acid, exhibited a T6SS-inhibiting role, protecting C6706Δ*tsiV1–3* from mucin-induced T6SS-killing. Taurodeoxycholic acid displayed weak T6SS inhibition.

To further investigate the modulating activities of bile acids, we took advantage of *V*. *cholerae* strain V52, which has a constitutive T6SS and does not rely on mucins for activation. As shown in Fig 4B, deoxycholic acid also inhibited the V52 T6SS. In contrast to the mucin-activated T6SS of C6706, glycodeoxycholic acid and taurodexoycholic acid stimulated the V52 T6SS at levels similar to unconjugated glycine and taurine. When deoxycholic acid was supplied in combination with either free glycine or taurine, an intermediate repression of T6SS activity was observed (Fig 4B). This suggests that the carboxylic acid group on deoxycholic acid (to which glycine and taurine are conjugated) is important for inhibition of the T6SS of *V*. *cholerae*. Activation of the T6SS by free glycine or taurine is a feature of bile acid conjugates and not a general property of amino acids, because the related amino acids alanine and phenylalanine had a slight negative effect on the T6SS of V52 (Fig 4B). We believe that this minor inhibitory phenotype is affecting a different mechanism of regulation than glycine and taurine, because the effects are different both in magnitude and direction. In conclusion, conjugation of either glycine or taurine to deoxycholic acid abolishes inhibition of the T6SS by deoxycholic acid.

### Deoxycholic acid downregulates the T6SS of *V*. *cholerae* by preventing tube formation

Next, we investigated how deoxycholic acid is able to downregulate the T6SS of *V*. *cholerae*. The T6SS of *C*. *jejuni*, an enteric pathogen that utilizes its T6SS for colonization, is also subject to downregulation by deoxycholic acid [60]. *C*. *jejuni* strains with an active T6SS display a higher susceptibility to the toxic effects of deoxycholic acid than those without a T6SS. Lertpiriyapong *et al*. speculated that the T6SS conduit allows diffusion of deoxycholic acid into the *C*. *jejuni* cell [60]. They went on to show that *C*. *jejuni* cells that survived the toxicity of deoxycholic acid adapted by down-regulating their T6SS [60]. Similar to the Lertpiriyapong *et al*. study [60], we performed growth assays using *V*. *cholerae* in liquid LB broth with increasing concentrations of deoxycholic acid (S3A Fig). We determined that toxicity did not increase for T6SS-positive bacteria compared to an isogenic mutant in which the T6SS was genetically disabled. In addition, we observed no deoxycholic acid-mediated toxicity when *V*. *cholerae* were grown on nutrient agar plates for our killing assay (S3B Fig), suggesting that the mechanism of down-regulation by deoxycholic acid of the T6SS is different than the adaption showed in *C*. *jejuni*.

**Fig 5:**
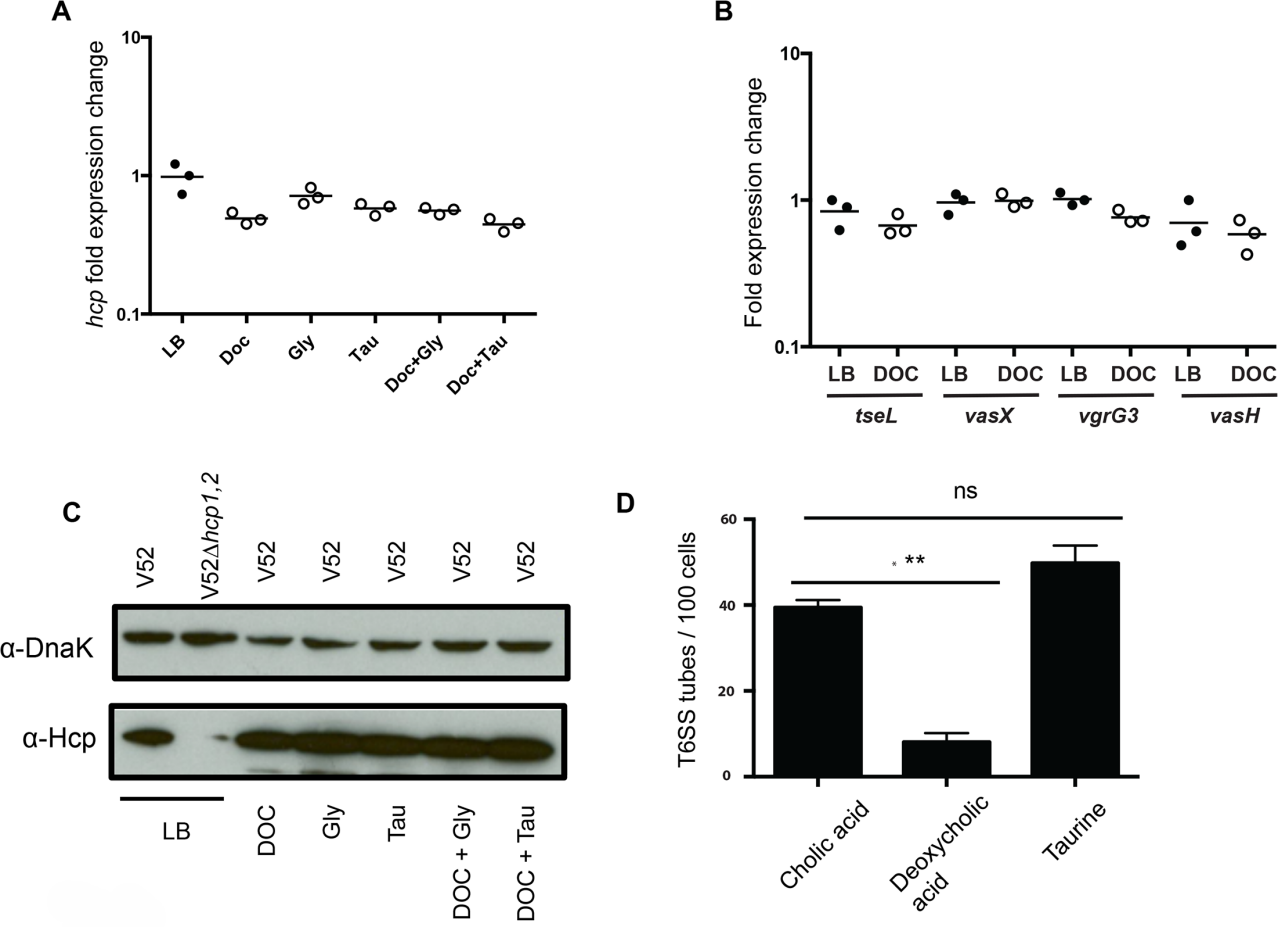
Deoxycholic acid regulates tube formation. **(A)** Bile salts do not affect the transcription of *hcp* in V52. V52 was incubated for two hours with 1.2 mM deoxycholic acid, glycine, taurine and combinations of the three. RNA was then isolated, converted to cDNA, quantified using qPCR, and compared to 16S rRNA gene. Experiments were performed in triplicates and normalized to the LB control. **(B)** Deoxycholic acid does not affect the transcription of *tseL*, *vasX*, *vgrG3*, *vasH* in V52. V52 was incubated with 1.2 mM deoxycholic acid for two hours. RNA was isolated, converted to cDNA and quantified using qPCR. Experiments were performed in triplicates and normalized to the LB control. **(C)** Bile salts do not affect Hcp-2 levels in V52. V52 was incubated with 1.2 mM deoxycholic acid, glycine, taurine and combinations of the three for two hours. Bacteria were harvested and western blot analysis was performed using antibodies for Hcp-2 and DnaK. **(D)** Deoxycholic acid affects the ability of the T6SS to form tubes. *V*. *cholerae* 2740–80 with a sfGFP labeled *vipA* was incubated with 1.2 mM cholate (negative control), deoxycholic acid or taurine for thirty minutes. After this incubation, the cells were imaged for ten minutes using the Super Resolution OMX microscope. Three frames were chosen and the number of extended T6SS tubes were counted. A Student’s *t*-test was performed for significance, with ** *p* < 0.005; ns is no significance.

To determine if deoxycholic acid regulates T6SS machinery on a transcriptional level, we first checked the transcript levels of hcp in bacteria grown in the absence or presence of various bile salts; we found that none of the bile salts changed Hcp transcript levels (Fig 5A). This matched data demonstrating that deoxycholic acid does not affect cell-associated Hcp protein levels (Fig 5C). We then performed qPCR to check for altered transcript levels of additional T6SS genes in the presence of deoxycholic acid. Again, we did not observe dramatic changes in mRNA levels for structural (*vasK*), regulatory (*vasH)*, or effector (*tseL*, *vasX*, and *vgrG3*) components (Fig 5B). This indicates that deoxycholic acid either affects gene expression of another T6SS gene or prevents T6SS complex formation.

To visualize the effect of deoxycholic acid on the T6SS, we utilized *V*. *cholerae* 2740–80 with an sfGFP labeled *vipA* and performed super-resolution microscopy to visualize the T6SS. *V*. *cholerae* 2740–80 is a nontoxigenic strain, which similar to V52 employs a constitutively active T6SS under laboratory conditions and has been used. We then incubated this bacterial strain on LB agar pads, LB pads mixed with deoxycholic acid, and LB pads mixed with taurine for 30 min before visualizing the T6SS. We determined the number of bacteria in the field of view and the number of T6SS tubes. We observed that tube formation in strains incubated on LB pads mixed with deoxycholic acid was reduced approximately 12.5-fold compared to bacteria incubated on plain LB pads (Fig 5D). This indicates that although T6SS-related genes do not appear to be down-regulated in the presence of deoxycholic acid, T6SS dynamics are decreased. We observed an insignificant increase in tube formation upon incubation with taurine. We hypothesize that the T6SS of 2740–80 is already hyperactive and unable to increase its activity. We hypothesize that deoxycholic acid regulates the T6SS by destabilizing the macromolecular tube complex, which prevents delivery of toxic effectors to neighboring cells.

### Commensal bacteria modify bile acids to abolish T6SS activity

During colonization of the small intestine, *V*. *cholerae* comes in close contact with the microbiota that inhabits the mucus layer [34]. Further, as *V*. *cholerae* is cleared from the small intestine in purges of watery diarrhea, *V*. *cholerae* enters the colon and comes in contact with other microbes. Many of these commensal bacteria, in the small intestine and the colon have the ability to modify bile acids via dehydroxylation or deconjugation [61] (Fig 3). *Bacteroides* and *Bifidobacterium* are among the major anaerobic bacteria in the gut that participate in bile acid metabolism [62–64]. As the microbiota can affect bile acid composition, and activity in the T6SS of *V*. *cholerae* is influenced by bile acids, we investigated the effect of microbiota bile acid metabolism on activation or inhibition of the T6SS.

**Fig 6:**
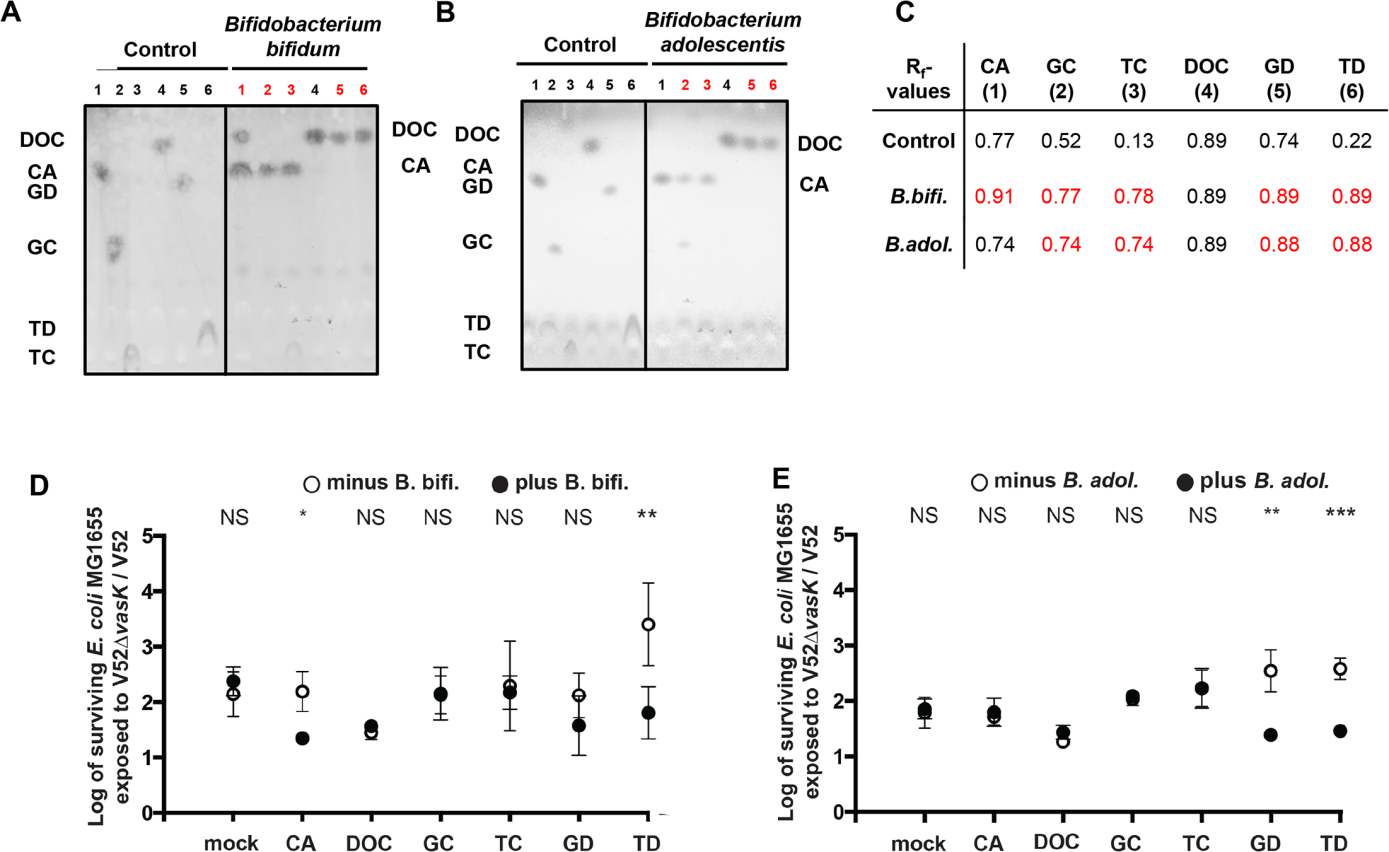
Commensal gut bacteria influence T6SS function. **(A)** Metabolism of bile acids by *B*. *bifidum*. TLC was performed with indicated bile acids in the presence or absence (control) of *B*. *bifidum*: cholic acid (CA), deoxycholic acid (DOC), glycocholic acid (GC), taurocholic acid (TC), glycodeoxycholic acid (GD), or taurodeoxycholic acid (TD), **(B)** Metabolism of bile acids by *B*. *adolescentis*. TLC was performed with indicated bile acids in the presence or absence (control) of *B*. *adolescentis*. **(C)** R_f_-values for TLC experiments. **(D)** *B*. *bifidum* metabolizes bile acids that lead to T6SS inhibition. *B*. *bifidum* was incubated under anaerobic conditions for 2 days on LB agar plates supplemented with 1.2 mM of one of the indicated bile acids. ‘Mock’ indicates the plates with no bile acids added. After removal of the anaerobes, *V*. *cholerae* V52 or V52Δ*vasK* were mixed at a 10:1 ratio with *E*. *coli* MG1655 and spotted on either on top of the removed commensal spot or 2 cm away from the commensal spot. Surviving *E*. *coli* bacteria were enumerated after 4 h-incubation at 37°C. Bars show mean values ± SD of two independent experiments done in triplicate. **(E)** *B*. *adolescentis* can metabolize GD and TD to inhibit the T6SS. *B*. *adolescentis* was incubated under anaerobic conditions for 2 days on LB agar plates supplemented with 1.2 mM of one of the indicated bile acids. ‘Mock’ indicates the plates with no bile acids added. After removal of the anaerobes, *V*. *cholerae* V52 or V52Δ*vasK* were mixed at a 10:1 ratio with *E*. *coli* MG1655 and spotted on top of the removed commensal spot or 2 cm away from the commensal spot. Surviving *E*. *coli* bacteria were enumerated after 4 h incubation at 37°C. Bars show mean values ± SD of two independent experiments done in triplicate. A Student’s *t*-test was performed for significance, with *p < 0.05, ** *p* < 0.005, *** *p* < 0.0005, ns = not significant.

The ability of four commensal bacterial species to modify bile acids and subsequently affect the T6SS activity of *V*. *cholerae* was analyzed by thin-layer chromatography (TLC) and killing assays. TLC separates bile acids based on their ability to bind to the stationary phase of a chromatography plate. Using purified bile acids as positive controls, we determined the ability of bacteria to convert one bile acid to another. We mixed individual bile acids with *Bifidobacterium bifidum or Bifidobacterium adolescentis* and ran the cell-free mixtures on TLC plates (Fig 6A, 6B & 6C). By comparing the resulting bands with our positive controls, we could see conversion of bile acids. For the killing assay, commensal bacteria of the species *Bacteroides thetaiotaomicron*, *B*. *bifidum*, *B*. *adolescentis*, and *Bifidobacterium subtile* were anaerobically grown on LB plates supplemented with either unconjugated or conjugated cholate or deoxycholate derivatives. After commensals were removed by chloroform vapor treatment [47], killing assays were performed under aerobic conditions on the pretreated plates with V52 or V52Δ*vasK* predator against prey *E coli* MG1655 (Fig 6D & 6E).

*B*. *bifidum*, a Gram-positive bacterium prevalent in the human intestine [65,66], dehydroxylated and deconjugated bile acids (Fig 6A & 6D). As shown by TLC, *B*. *bifidum* deconjugated glycodeoxycholic or taurodeoxycholic acids to deoxycholic acid, and glycocholic or taurocholic acids to cholic acid. In addition, *B*. *bifidum* dehydroxylated cholic to deoxycholic acid. T6SS-mediated killing of *E*. *coli* on plates supplemented with glycodeoxycholic or taurodeoxycholic acids and previously treated with *B*. *bifidum* (Fig 6D) was reduced compared to plates not treated with *B bifidum*. As expected, conversion of glycocholic or taurocholic acids to cholic acid had no effect on the T6SS. A similar inhibition of T6SS-mediated killing was observed on plates supplemented with cholic acid and previously treated with *B*. *bifidum*, because cholic acid is dehydroxylated to deoxycholic acid in the presence of *B*. *bifidum*. The killing activity decreased to a level comparable to that observed in the presence of deoxycholic acid (the end product of glycodeoxycholic acid or taurodeoxycholic acid deconjugation and cholic acid dehydroxylation). We conclude that *B*. *bifidum* negatively regulates T6SS activity through the metabolic conversion of each of three bile acids, glycodeoxycholic, taurodeoxycholic, or cholic acid, to deoxycholic acid.

Similarly to *B*. *bifidum*, the two commensals *B*. *adolescentis* and *B*. *subtile* also modified the effects of selected bile acids on T6SS activity. *B*. *adolescentis* and *B*. *bifidum* both deconjugate glycocholic or taurocholic acids to cholic acid, and glycodeoxycholic or taurodeoxycholic acids to deoxycholic acid. However, *B*. *adolescentis* is unable to dehydroxylate cholic to deoxycholic acid. We conclude that the decrease in T6SS activity (Fig 6E) was due to the deconjugation of glycodeoxycholic or taurodeoxycholic acids to deoxycholic acid by *B*. *adolescentis* (Fig 6A and 6B). *B*. *subtile* has a more specific metabolic activity and can only deconjugate glycodeoxycholic acid to deoxycholic acid (S4A Fig and S4B Fig), resulting in a decrease in T6SS activity (S4E Fig). The reduced *V*. *cholerae* killing activity on LB plates previously treated with *B*. *adolescentis* and *B*. *subtile* was analogous to what was observed in plates previously treated with *B*. *bifidobacterium*, suggesting that reduced killing is due to the bile acid-converting activities of commensals.

The last commensal analyzed, *B*. *thetaiotaomicron*, did not deconjugate or dehydroxylate any of the bile acids as determined by TLC (S4A Fig and S4D Fig). However, the killing of *E*. *coli* was marginally inhibited on plates containing glycodeoxycholic acid and treated with *B*. *thetaiotaomicron* (S4D Fig). This observation could be explained by trace amounts of glycodeoxycholic acid conversion to deoxycholic acid undetected by TLC.

We conclude that metabolic products of bile acids produced by the host inhibit the T6SS of *V*. *cholerae*.

## Discussion

The marine bacterium *V*. *cholerae* thrives in a wide variety of environments and has evolved mechanisms to sense cues that control host colonization and virulence factors in a spatiotemporal fashion. Together with the recent findings of the Mekalanos group, our *in-vivo* experiments demonstrate the importance of the host environment in the T6SS activation of pandemic *V cholerae* strains (Fig 1) [27,29]. Host factors responsible for *in-vivo* activation of T6SS for pandemic strains were unknown. Our finding that the T6SS is functional under anaerobic conditions (S1C Fig) prompted us to identify host cues for T6SS activation and to investigate the role of anaerobic commensal bacteria in regulating the T6SS. Our findings that mucins activate the T6SS and that activation by mucins can further be modulated by bile acids under the metabolic control of commensal microbiota provide new insights into the complex regulation of the T6SS *in vivo*. We hypothesize that the activated T6SS is used by *V*. *cholerae* to counteract host defense cells and to compete with other bacteria for nutrients and space during infection of the host small intestine. These other bacteria could be commensals, other pathogens, or members of the same species.

V52 and C6706 regulate their T6SS differently. V52 employs a constitutively active T6SS, whereas C6706 has a repressed T6SS that is activated *in vivo*. Diversity of T6SS regulation among *V*. *cholerae* strains might indicate a diversity of biological function between pandemic strains and those associated with smaller outbreaks. T6SS regulators are likely utilized differently in V52 and C6706. If bile acids target these regulators, modulation of the T6SS by bile acids would be strain-dependent as observed in Fig 6 and S4 Fig. C6706 and other pandemic strains have a T6SS that is repressed under laboratory conditions and potentially in other stages of the lifecycle. However, as shown recently for chitin and now for mucins, pandemic *V*. *cholerae* strains can de-repress their T6SS to compete within their species and with other prokaryotes [31]. *V*. *cholerae* is often introduced to the human host by the ingestion of contaminated water, thus multiple strains may launch an infection. Activation of the T6SS in pandemic strains by mucins may allow the pandemic *V*. *cholerae* to kill competing *V*. *cholerae* strains to become the dominant agent of infection.

Our findings suggest that products of bile acid metabolism in commensal bacteria have roles in regulating *V*. *cholerae* virulence factors. Such effects would be expected to differ among human hosts depending on host microbiota composition. Hsaio et al. recently shed light on how members of the microbiota modulate *V*. *cholerae* infection [48]. They showed that different members of the human microbiota were able to prevent *V*. *cholerae* infection. Our findings identified additional members of the human microbiota to develop host-based therapies that minimize the effects of a *V*. *cholerae* infection.

There is precedence for a role for bile in controlling pathogen persistence. Fecal microbiota transplantation was recently proposed to prevent recurrence of *C*. *difficile* infection by correcting bile acid metabolism [67]. Thus, experimental support is emerging for the idea that the host microbiota composition determines the course of *C*. *difficile* infection [68,69]. Furthermore, modification of host factors such as bile and mucins might help a microbiota adapt to defend itself against pathogenic bacteria. Alternatively, as the commensal organisms utilized in this study were mainly colonic, *V*. *cholerae* may utilize bile salts as a spatial signal such that it recognizes deoxycholic acid as a signal to turn off its T6SS in the colon before being released into the environment. Locations in the gut where bile-deconjugating and-dehydroxylating commensals are absent may experience *V*. *cholerae* bacteria with higher T6SS activity than in locations where these commensal species are present. Therefore, preventive alteration of the microbiota (through the addition of *Bifidobacterium)* in people living in areas where cholera is endemic may disrupt *V*. *cholerae’s* T6SS, leading to a less fit organism and a reduction in disease outcome. Although our work does not demonstrate a necessity for the T6SS in colonization of the infant mouse, we do see competition indicating a role for the T6SS in intraspecies competition during a multi-strain infection. To test the efficacy of these probiotics, we suggest using the adult mouse model of cholera infection [70,71]. The adult mouse model can be used to study this hypothesis over a longer course of infection and in the presence of bile, as bile is believed to be absent from the infant mouse [65].

As commensal organisms such as *Bifidobacterium* and *Bacteriodes* are lost through diarrheal purges during acute *V*. *cholerae* infection [62], the levels of bile acids repressing the T6SS might decrease. This would allow the pathogen to utilize its T6SS in a re-infection of patients recovering from a recent cholera episode, or during later stages of the purge, providing an opportunity for new therapies that introduce *Bifidobacterium* species at an early stage of *V*. *cholerae* infection to decrease pathogen T6SS activity.

In conclusion, *V*. *cholerae* infection is complex and involves host factors such as bile, mucins, and a microbiota that have impact on the pathogen and regulation of its T6SS. This work describes a novel mechanism for two-pronged regulation of a virulence pathway in *V*. *cholerae*, through mucins that activate the pathway and bile metabolites that repress it. This work adds to our understanding of how pandemic *V*. *cholerae* strains have evolved as such successful pathogens.

## Supporting Information

S1 Fig Characterizing the T6SS of *V*. *cholerae in vivo* and under anaerobic conditions. **(A)** The T6SS of *V*. *cholerae* O1 strain C6706 is not required to grow to high titers in the small intestine of infant mice. We mixed *V*. *cholerae* C6706 with C6706Δ*lacZ*, or C6706*ΔlacZΔvasK* with C6706*ΔtsiV1-3ΔlacZ* and administered the mixtures to the infant mouse via oral gavage. As an *in-vitro* control, we added strain mixtures to 2 mL LB to be grown overnight at 37°C. After a 16-h incubation, the mice were sacrificed, their small intestines were harvested and plated on X-gal plates to count the total numbers of surviving bacteria. **(B)** The T6SS of *V*. *cholerae* O1 strain C6706 is not involved in the colonization of the infant mouse model of infection. We mixed *V*. *cholerae* C6706*ΔlacZΔvasK* and C6706*ΔvasKΔtsiV1-3* and administered to the infant mouse via oral gavage. As an *in-vitro* control, we added strain mixtures to 2 mL LB to be grown overnight at 37°C. After a 16-h incubation, the mice were sacrificed, their small intestines were harvested and plated on X-gal plates to count surviving bacteria The competitive index of the two competing strains is shown on the *y-*axis. Horizontal bars represent the geometric mean of one experiment performed with a minimum of 6 mice in each group. **(C)** Anaerobic conditions do not affect the T6SS activity of *V*. *cholerae*. We mixed *V*. *cholerae* V52, V52Δ*vasK*, or C6706 at a 10:1 ratio with *E*. *coli* MG1655 and employed in a killing assay under anaerobic or aerobic conditions. After a 4-h incubation at 37°C, numbers of surviving *E*. *coli* bacteria were plotted. Bars show mean values ± SD of two independent experiments done in duplicate. A Student’s *t*-test was performed for significance, with ***p < 0.0005, ns = not significant.
(TIF)

S2 Fig The absence of *tsiV1-3* does not affect the viability of C6706 in the presence of various bile salts. Growth curves of C6706 and C6706Δ*tsiV1-3* in various bile salts. A 1:100 dilution of overnight culture was made in LB, and LB with cholic acid (CA), glycholic acid (GC), taurocholic acid (TC), deoxycholic acid (DOC), glycodeoxycholic acid (GD), taurodeoxycholic acid (TD) glycine (Gly) or taurine (Tau). OD_600_ values were measured for both *V*. *cholerae* mutants under these conditions every 60 min for 4h. The slope of each growth curve was calculated for mid-log bacteria. The slope of C6706 was divided by C6706Δ*tsiV1-3* and plotted.
(TIF)

S3 Fig Deoxycholic acid (DOC), glycine and taurine affect the T6SS of *V*. *cholerae*. **(A)** Growth curves of V52 and V52Δ*vasK* in various concentrations of DOC. A 1:100 dilution of overnight culture was made in LB, LB + 0.005% DOC, LB + 0.05% DOC and LB + 0.5% DOC. OD_600_ values were measured for both *V*. *cholerae* strains under these four concentrations every 30 min for 6 h. The slope of each growth curve was calculated for mid-log bacteria. The slopes of the growth curves in LB + DOC were compared to the slopes in LB alone and the ratios were plotted. **(B)** Killing assays in the presence of various concentrations of DOC. *V*. *cholerae* V52 or V52Δ*vasK* were mixed at a 10:1 ratio with *E*. *coli* MG1655 and employed in a killing assay with *E*. *coli* MG1655 as prey. Surviving numbers of *E*. *coli* are plotted on the y-axis. Bars show mean values ± SD of two independent experiments done in duplicate. **(C)** Killing assays in the presence of various concentrations of taurine and glycine. *V*. *cholerae* V52 or V52Δ*vasK* were mixed at a 10:1 ratio with *E*. *coli* MG1655 and employed in a killing assay with *E*. *coli* MG1655 as prey. Surviving numbers of *E*. *coli* are plotted on the graph. Bars show mean values ± SD of two independent experiments done in duplicate. **(D)** DOC inhibits the T6SS of *V*. *cholerae* C6706Δ*luxO*Δ*tsrA*. Predator *V*. *cholerae* C6706, or C6706 with deletions in *luxO* and *tsrA* (C6706Δ*luxO*Δ*tsrA*) to generate a functional T6SS, were mixed with prey *E*. *coli* MG1655 and subjected to a killing assay as described in (B). A killing index was calculated as the ratio of surviving prey in the presence of C6706Δ*luxO*Δ*tsrA* to surviving prey in the presence of C6706. The graph gives mean values ± SD of two experiments done in duplicate. A Student’s *t*-test was performed for significance, with ***p < 0.0005 and ns = not significant.
(TIF)

S4 Fig Commensal gut bacteria can influence T6SS function. **(A)** Metabolism of bile acids by *B*. *thetaiotaomicron*. TLC was performed with indicated bile acids in the presence or absence (control) of *B*. *thetaiotaomicron*: cholic acid (CA), deoxycholic acid (DOC), glycocholic acid (GC), taurocholic acid (TC), glycodeoxycholic acid (GD), or taurodeoxycholic acid (TD), **(B)** Metabolism of bile acids by *B*. *subtile*. TLC was performed with indicated bile acids in the presence or absence (control) of *B*. *subtile*. **(C)** R_f_-values for TLC experiments. **(D)** *B*. *thetaiotaomicron* does not metabolize bile acids to affect the T6SS. *B*. *thetaiotaomicron* was incubated under anaerobic conditions for 2 days on LB agar plates supplemented with 1.2 mM of one of the indicated bile acids. ‘Mock’ indicates the killing assay with no added bile acids. After removal of the anaerobes, *V*. *cholerae* V52 or V52Δ*vasK* were mixed at a 10:1 ratio with *E*. *coli* MG1655 and spotted on one plate either on top of the removed commensal spot or 2 cm away from the commensal spot. Surviving *E*. *coli* bacteria were enumerated after 4 h incubation at 37°C. Bars show mean values ± SD of two independent experiments done in triplicate. **(E)** *B*. *subtile* metabolizes taurodeoxycholic acid to inhibit the T6SS. *B*. *subtile* was incubated under anaerobic conditions for 2 days on LB agar plates supplemented with 1.2 mM of one of the indicated bile acids. ‘Mock’ indicates the killing assay with no added bile acids. After removal of the anaerobes, *V*. *cholerae* V52 or V52Δ*vasK* were mixed at a 10:1 ratio with *E*. *coli* MG1655 and spotted on one plate either on top of the removed commensal spot or 2 cm away from the commensal spot. Surviving *E*. *coli* bacteria were enumerated after 4 h incubation at 37°C. Bars show mean values ± SD of two independent experiments done in triplicate. A Student’s *t*-test was performed for significance, with *p < 0.05, ns = not significant.
(TIF)
