# Supplementary figures and images for: Bile Salts Modulate the Mucin-Activated Type VI Secretion System of Pandemic Vibrio cholerae
Source: PLoS Negl Trop Dis. 2015 Aug 28;9(8):e0004031. doi: 10.1371/journal.pntd.0004031 (PMC4552747; doi:10.1371/journal.pntd.0004031)

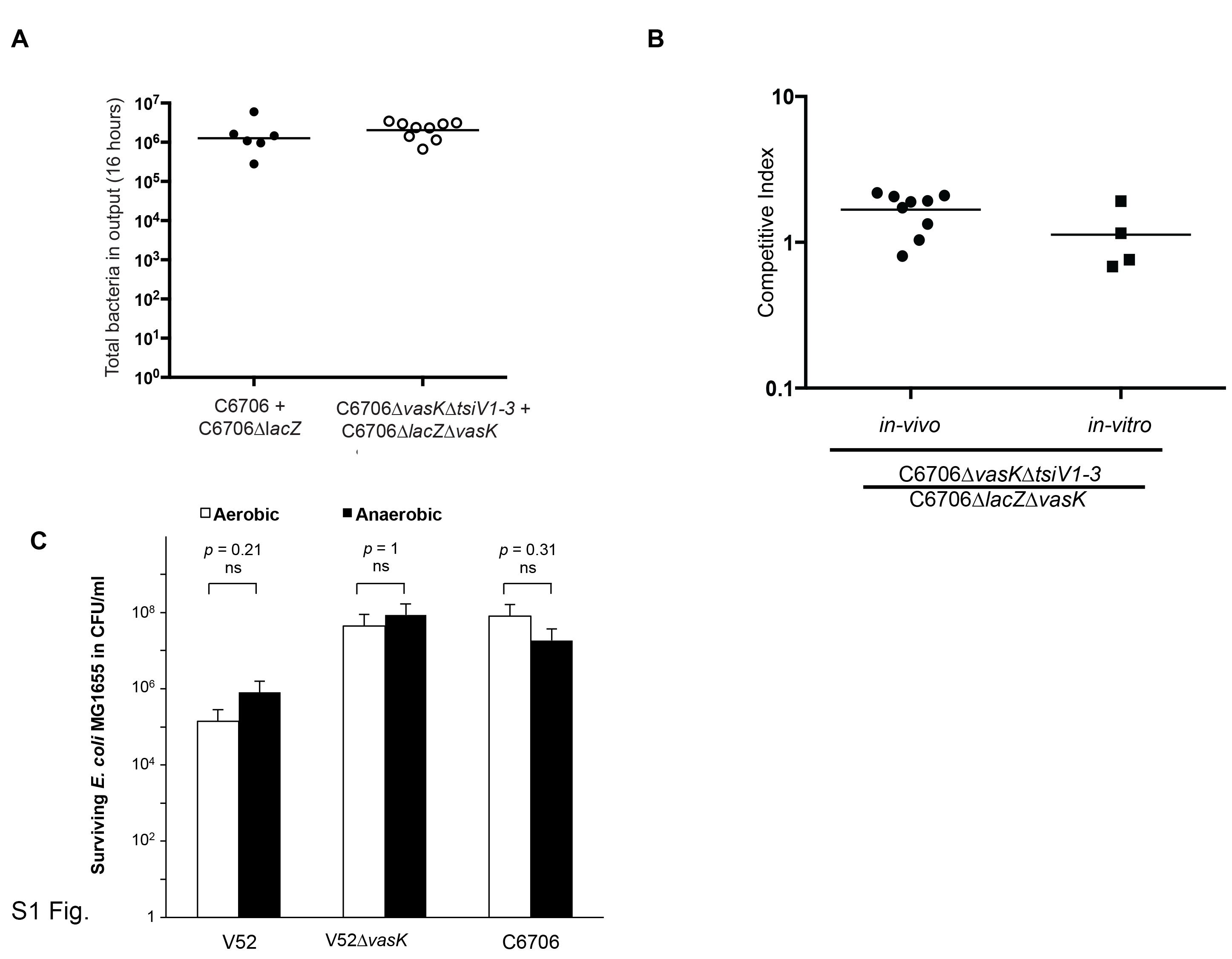

Supplement: S1 Fig — (A) The T6SS of V. cholerae O1 strain C6706 is not required to grow to high titers in the small intestine of infant mice. We mixed V. cholerae C6706 with C6706ΔlacZ, or C6706ΔlacZΔvasK with C6706ΔtsiV1-3ΔlacZ and administered the mixtures to the infant mouse via oral gavage. As an in-vitro control, we added strain mixtures to 2 mL LB to be grown overnight at 37°C. After a 16-h incubation, the mice were sacrificed, their small intestines were harvested and plated on X-gal plates to count the total numbers of surviving bacteria. (B) The T6SS of V. cholerae O1 strain C6706 is not involved in the colonization of the infant mouse model of infection. We mixed V. cholerae C6706ΔlacZΔvasK and C6706ΔvasKΔtsiV1-3 and administered to the infant mouse via oral gavage. As an in-vitro control, we added strain mixtures to 2 mL LB to be grown overnight at 37°C. After a 16-h incubation, the mice were sacrificed, their small intestines were harvested and plated on X-gal plates to count surviving bacteria The competitive index of the two competing strains is shown on the y-axis. Horizontal bars represent the geometric mean of one experiment performed with a minimum of 6 mice in each group. (C) Anaerobic conditions do not affect the T6SS activity of V. cholerae. We mixed V. cholerae V52, V52ΔvasK, or C6706 at a 10:1 ratio with E. coli MG1655 and employed in a killing assay under anaerobic or aerobic conditions. After a 4-h incubation at 37°C, numbers of surviving E. coli bacteria were plotted. Bars show mean values ± SD of two independent experiments done in duplicate. A Student’s t-test was performed for significance, with ***p < 0.0005, ns = not significant. (TIF) [file pntd.0004031.s001.tif]

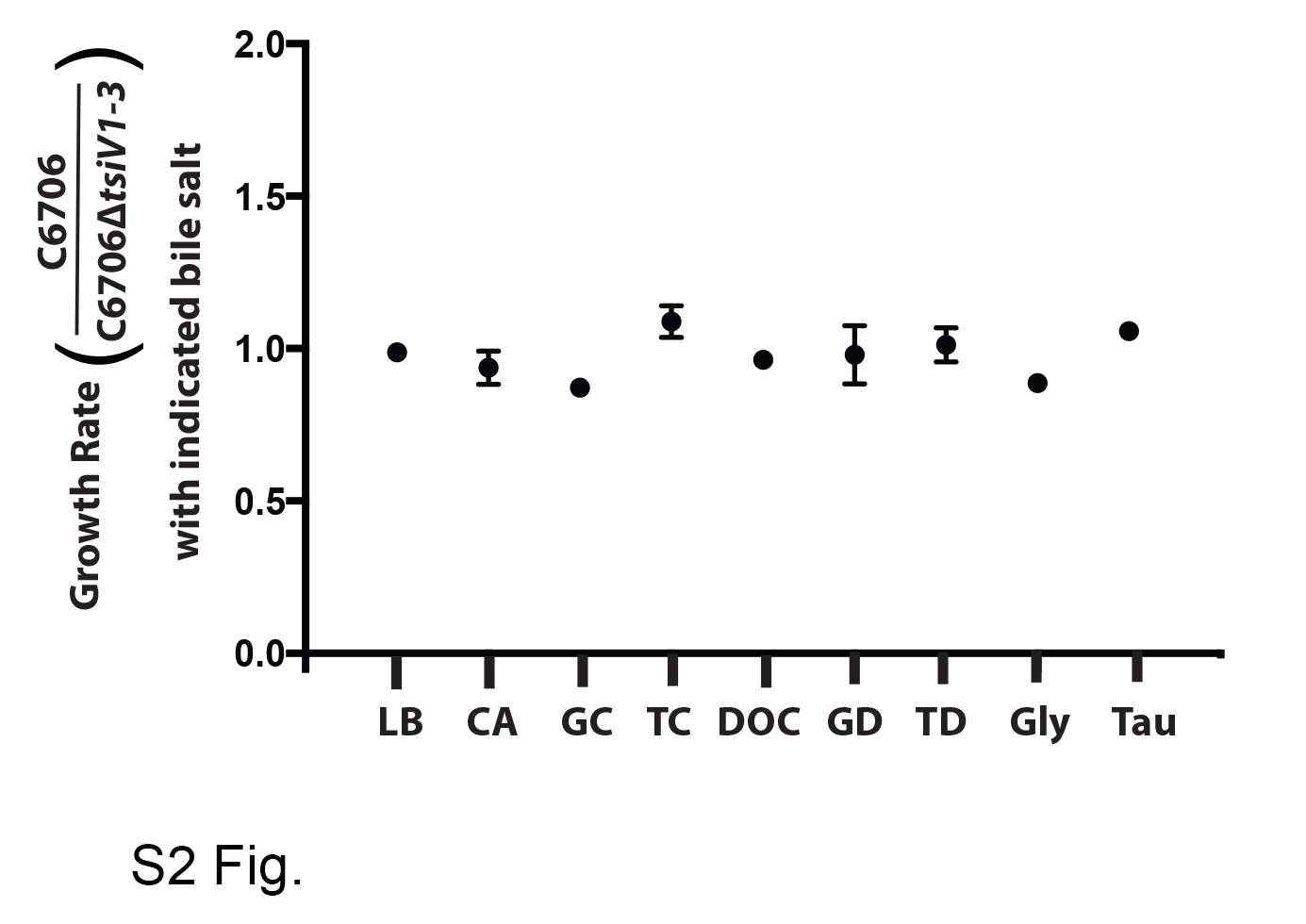

Supplement: S2 Fig — Growth curves of C6706 and C6706ΔtsiV1-3 in various bile salts. A 1:100 dilution of overnight culture was made in LB, and LB with cholic acid (CA), glycholic acid (GC), taurocholic acid (TC), deoxycholic acid (DOC), glycodeoxycholic acid (GD), taurodeoxycholic acid (TD) glycine (Gly) or taurine (Tau). OD600 values were measured for both V. cholerae mutants under these conditions every 60 min for 4h. The slope of each growth curve was calculated for mid-log bacteria. The slope of C6706 was divided by C6706ΔtsiV1-3 and plotted. (TIF) [file pntd.0004031.s002.tif]

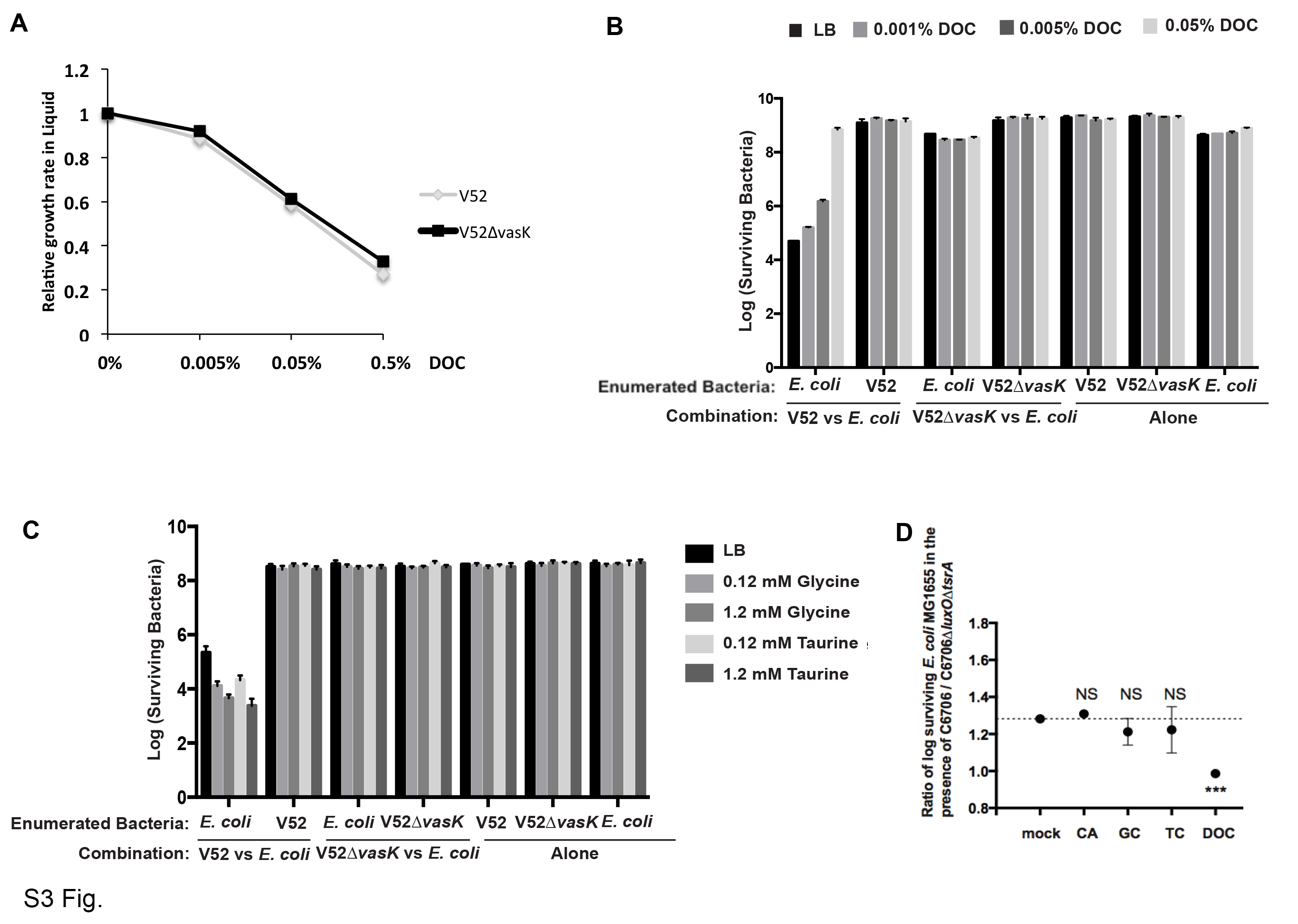

Supplement: S3 Fig — (A) Growth curves of V52 and V52ΔvasK in various concentrations of DOC. A 1:100 dilution of overnight culture was made in LB, LB + 0.005% DOC, LB + 0.05% DOC and LB + 0.5% DOC. OD600 values were measured for both V. cholerae strains under these four concentrations every 30 min for 6 h. The slope of each growth curve was calculated for mid-log bacteria. The slopes of the growth curves in LB + DOC were compared to the slopes in LB alone and the ratios were plotted. (B) Killing assays in the presence of various concentrations of DOC. V. cholerae V52 or V52ΔvasK were mixed at a 10:1 ratio with E. coli MG1655 and employed in a killing assay with E. coli MG1655 as prey. Surviving numbers of E. coli are plotted on the y-axis. Bars show mean values ± SD of two independent experiments done in duplicate. (C) Killing assays in the presence of various concentrations of taurine and glycine. V. cholerae V52 or V52ΔvasK were mixed at a 10:1 ratio with E. coli MG1655 and employed in a killing assay with E. coli MG1655 as prey. Surviving numbers of E. coli are plotted on the graph. Bars show mean values ± SD of two independent experiments done in duplicate. (D) DOC inhibits the T6SS of V. cholerae C6706ΔluxOΔtsrA. Predator V. cholerae C6706, or C6706 with deletions in luxO and tsrA (C6706ΔluxOΔtsrA) to generate a functional T6SS, were mixed with prey E. coli MG1655 and subjected to a killing assay as described in (B). A killing index was calculated as the ratio of surviving prey in the presence of C6706ΔluxOΔtsrA to surviving prey in the presence of C6706. The graph gives mean values ± SD of two experiments done in duplicate. A Student’s t-test was performed for significance, with ***p < 0.0005 and ns = not significant. (TIF) [file pntd.0004031.s003.tif]

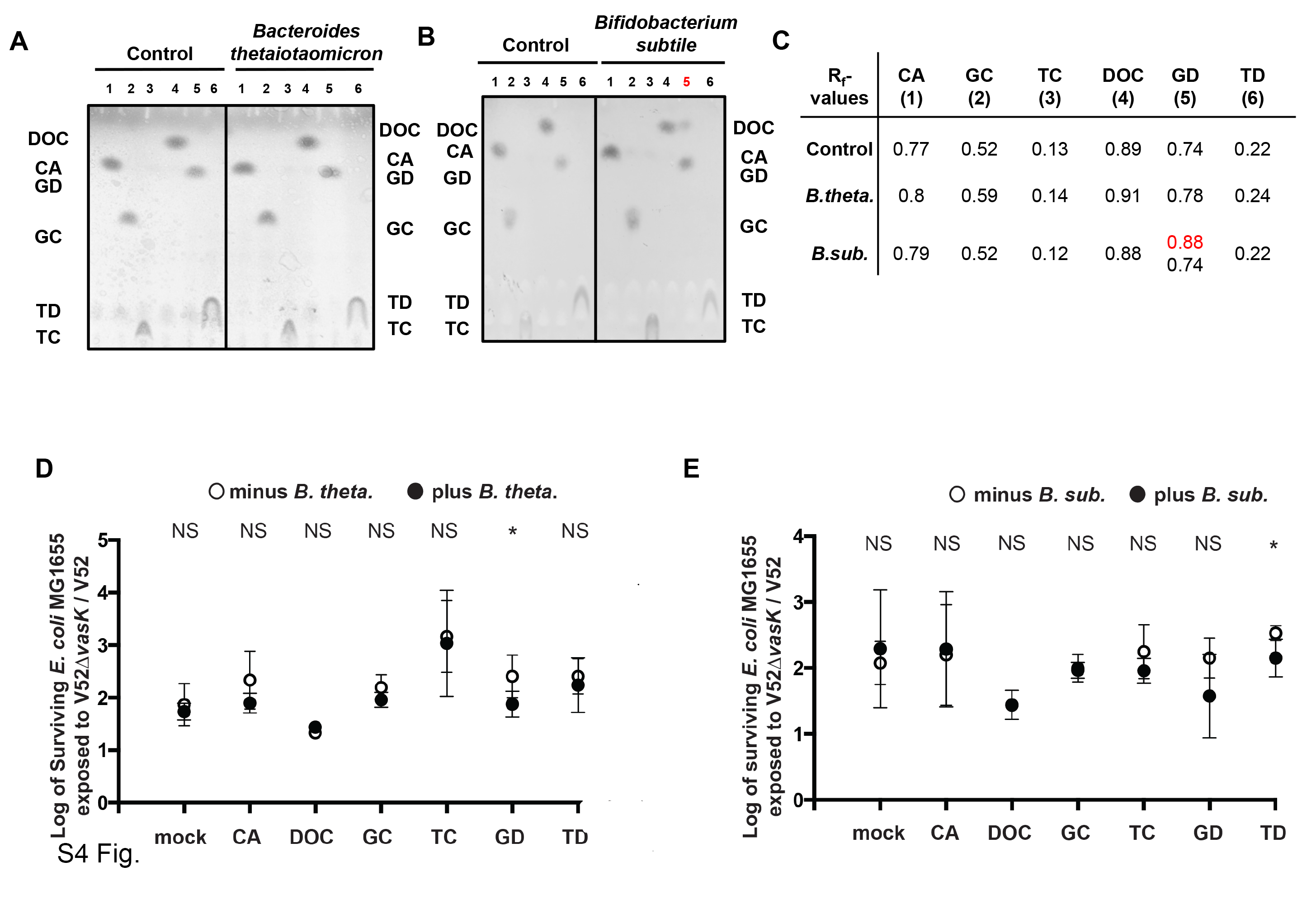

Supplement: S4 Fig — (A) Metabolism of bile acids by B. thetaiotaomicron. TLC was performed with indicated bile acids in the presence or absence (control) of B. thetaiotaomicron: cholic acid (CA), deoxycholic acid (DOC), glycocholic acid (GC), taurocholic acid (TC), glycodeoxycholic acid (GD), or taurodeoxycholic acid (TD), (B) Metabolism of bile acids by B. subtile. TLC was performed with indicated bile acids in the presence or absence (control) of B. subtile. (C) Rf-values for TLC experiments. (D) B. thetaiotaomicron does not metabolize bile acids to affect the T6SS. B. thetaiotaomicron was incubated under anaerobic conditions for 2 days on LB agar plates supplemented with 1.2 mM of one of the indicated bile acids. ‘Mock’ indicates the killing assay with no added bile acids. After removal of the anaerobes, V. cholerae V52 or V52ΔvasK were mixed at a 10:1 ratio with E. coli MG1655 and spotted on one plate either on top of the removed commensal spot or 2 cm away from the commensal spot. Surviving E. coli bacteria were enumerated after 4 h incubation at 37°C. Bars show mean values ± SD of two independent experiments done in triplicate. (E) B. subtile metabolizes taurodeoxycholic acid to inhibit the T6SS. B. subtile was incubated under anaerobic conditions for 2 days on LB agar plates supplemented with 1.2 mM of one of the indicated bile acids. ‘Mock’ indicates the killing assay with no added bile acids. After removal of the anaerobes, V. cholerae V52 or V52ΔvasK were mixed at a 10:1 ratio with E. coli MG1655 and spotted on one plate either on top of the removed commensal spot or 2 cm away from the commensal spot. Surviving E. coli bacteria were enumerated after 4 h incubation at 37°C. Bars show mean values ± SD of two independent experiments done in triplicate. A Student’s t-test was performed for significance, with *p < 0.05, ns = not significant. (TIF) [file pntd.0004031.s004.tif]
